# A prospective study of cellular immune response to booster COVID-19 vaccination in multiple sclerosis patients treated with a broad spectrum of disease-modifying therapies

**DOI:** 10.1007/s00415-023-11575-8

**Published:** 2023-03-18

**Authors:** Pascual Torres, Agustín Sancho-Saldaña, Anna Gil Sánchez, Silvia Peralta, Maria José Solana, Sofian Bakkioui, Cristina González-Mingot, Laura Quibus, Emilio Ruiz-Fernández, Eduardo San Pedro-Murillo, Luis Brieva

**Affiliations:** 1 Metabolic Pathophysiology Research Group, Department of Experimental Medicine, University of Lleida (UdL)-IRBLleida, 25198 Lleida, Spain; 2 Neuroimmunology Group, Department of Medicine, University of Lleida (UdL)-IRBLleida, 25198 Lleida, Spain; 3 Department of Neurology, Hospital Universitari Arnau de Vilanova, 25198 Lleida, Spain

**Keywords:** Multiple sclerosis, Cellular immune response, COVID-19 vaccines, Immunomodulating drugs, Booster revaccination

## Abstract

**Background:**

Most people with Multiple Sclerosis (pwMS) are subjected to immunomodulatory disease-modifying treatments (DMTs). As a result, immune responses to COVID-19 vaccinations could be compromised. There are few data on cellular immune responses to the use of COVID-19 vaccine boosters in pwMS under a broad spectrum of DMTs.

**Methods:**

In this prospective study, we analysed cellular immune responses to SARS-CoV-2 mRNA booster vaccinations in 159 pwMS with DMT, including: ocrelizumab, rituximab, fingolimod, alemtuzumab, dimethyl fumarate, glatiramer acetate, teriflunomide, natalizumab and cladribine.

**Results:**

DMTs, and particularly fingolimod, interact with cellular responses to COVID-19 vaccination. One booster dose does not increase cellular immunity any more than two doses, except in the cases of natalizumab and cladribine. SARS-CoV-2 infection combined with two doses of vaccine resulted in a greater cellular immune response, but this was not observed after supplementary booster jabs. Ocrelizumab-treated pwMS who had previously received fingolimod did not develop cellular immunity, even after receiving a booster. The time after MS diagnosis and disability status negatively correlated with cellular immunity in ocrelizumab-treated pwMS in a booster dose cohort.

**Conclusions:**

After two doses of SARS-CoV-2 vaccination, a high response yield was achieved, except in patients who had received fingolimod. The effects of fingolimod on cellular immunity persisted for more than 2 years after a change to ocrelizumab (which, in contrast, conserved cellular immunity). Our results confirmed the need to find alternative protective measures for fingolimod-treated people and to consider the possible failure to provide protection against SARS-CoV-2 when switching from fingolimod to ocrelizumab.

## Introduction

COVID-19 is a respiratory disease caused by SARS-CoV-2 infection which has a wide range of symptoms. The COVID-19 pandemic has generated an emergence in health systems worldwide. In Spain, which was one of the most affected countries at the beginning of the pandemic, hospital capacities were often exceeded as mortality rose shapely [1]. COVID-19 mortality is strongly associated with age and cardiovascular and respiratory comorbidities [2]. People with Multiple Sclerosis (pwMS) and comorbidities have been treated with anti-CD20, used as a Disease-Modifying Therapy (DMT). When they received methylprednisolone in the previous month, they exhibited a greater risk of contracting severe COVID-19 [3, 4]. We have previously reported an increased susceptibility to SARS-CoV-2 infection in pwMS. However, in the previous study, severity was no different from in the general population. Interestingly, interferon intake was significantly associated with the presence of a greater presence of SARS-CoV-2 antibodies, which was probably due to a better anti-viral response [5]. Notwithstanding this, an independent group found an increased risk of severe COVID-19 in pwMS when they were treated with an anti-CD20 agent (ocrelizumab or rituximab) DMT [6].

Fortunately, COVID-19 vaccines have dramatically changed the course of the pandemic. A mathematical model has predicted that the use of vaccines averted 19.8 million deaths in the first year of vaccination. It has been estimated that 31.4 million deaths would have occurred without vaccination. Worldwide, vaccination has, therefore, probably reduced total deaths due to COVID-19 by 63% (19.8 million of 31.4 million) in one year, albeit with considerable differences between high-income and low-income countries [7]. Most countries give priority to the most vulnerable sectors of the population (the elderly and/or those with risk-associated comorbidities) for vaccination. The emergence of immune evasive strains, such as Omicron variants, has accelerated the administration of booster doses. The pandemic is still on-going and the evolution of SARS-CoV-2 has called for the development of more vaccine strains [8]. Within this context, the role of the immunocompromised (such as pwMS) in promoting novel SARS-CoV-2 immune escape mutations has been of particular concern [9].

COVID-19 vaccines stimulate two immune responses: humoral and cellular. On the one hand, humoral immunity is based on neutralizing the production of antibodies (nAb) by plasma B cells. On the other, cellular, or cell-mediated, immunity involves the release of cytokines and the activation of phagocytes and cytotoxic T cells that can remove infected cells. Population-based surveys analyse only humoral immunity [10]. However, memory T cells (the main component of cellular immunity), whether generated by vaccination or infection, recognize the less frequently mutated epitopes associated with Omicron. Cellular immunity is, therefore, conserved in the case of novel strains, while nAbs efficiency is dramatically reduced in that of Omicron [11]. Public health should focus on monitoring cell-mediated immunity for long-term surveys because new variants are constantly emerging and often escape nABs. Moreover, cellular immunity is maintained for at least 17 years in the case of SARS-CoV-1 and generates multi-specific immunity against other betacoronaviruses [12]. In contrast, the number of nAbs generated by vaccination peaks after 1 week, but is reduced to the baseline value of 1 dose after 6 months [13]. Furthermore, the early induction of cellular immunity is present in patients with mild cases of COVID-19 and accelerates viral clearance, while titters of SARS-CoV-2-specific antibodies are greater in cases of severe COVID-19 [14].

Most of the studies involving pwMS carried out to date have measured the humoral response to vaccination and ignored the cellular response [15]. Current knowledge about the interaction between DMTs and vaccine efficacy points to a reduced humoral response to anti-CD20 (ocrelizumab and rituximab) and fingolimod. However, pwMS under treatment with ocrelizumab develop a cellular response after vaccination [16, 17]. We hypothesize that a third COVID-19 vaccination dose boosts cellular immunity in pWMS treated with ocrelizumab, rituximab, cladribine, glatiramer acetate, teriflunomide, dimethyl fumarate and alemtuzumab but not those receiving fingolimod.

In this study, we sought to analyse the cellular immunity of pwMS both before and after receiving a booster dose of COVID-19 vaccine. We found a > 75% positive response after > 6 months of receiving two doses of SARS-CoV-2 vaccine for those receiving natalizumab, ocrelizumab, cladribine and teriflunomide; 50–75% for those treated with rituximab, alemtuzumab, glatiramer acetate and dimethyl fumarate; and 25% in the case of patients receiving fingolimod. Surprisingly, the booster doses had only a limited effect on cellular immunity in most of the DMT groups. Previous SARS-CoV-2 infection induced a greater cellular immune response when this was combined with vaccination including two doses of SARS-CoV-2 vaccine, but this effect was diluted after receiving a booster. Interestingly, previous fingolimod administration induced a weaker response in the ocrelizumab group. Disease evolution (time after diagnosis and disability) had a negative correlation with cellular immune response to the booster dose in patients treated with ocrelizumab.

## Methods

### Study design and patients

This is a single-centre, prospective, observational study which aimed to determine cellular response both before and after patients received a booster (third) dose of SARS-CoV-2 vaccine. In total, 159 participants were enrolled in the study, which was conducted between November 2021 and June 2022. All of the participants had been diagnosed with MS, based on the McDonald criteria of 2017, [18]. They had all been treated with DMTs, had not had disease activity (relapses) for at least 3 months, had not previously received glucocorticoids, for at least 3 months, and their last anti-CD20 cycles had been more than 5 months prior to the date of sample collection.

Recruitment was performed by *Consultes Externes de Neurologia* at the Hospital Universitari Arnau de Vilanova (HUAV) in Lleida and the study was approved by the local ethics committee. Informed consent was obtained from all the participants. The follow-up period after the booster was 2.8 ± 1.7 (0.2–8.4) months. Baseline characteristics (sex, age, MS type and EDSS), MS history (time from MS diagnosis, and previous and current DMTs), Absolute Lymphocyte Count (ALC), and symptoms of COVID-19 were recorded, and correlations between any of these characteristics and the cellular immune response to SARS-CoV-2 specific antigen in blood samples were analysed.

The last fingolimod dose received by those in the ocrelizumab group had been taken 0.95–2.01 years before the first vaccine dose. The corresponding periods were 3.3 years for alemtuzumab, 1.61 for cladribine, and 1.55–6.04 for rituximab. In all cases, the washout period was two months and the ALC was > 500 cells/μl.

PwMS with positive Antigen Test or PCR results were considered confirmed cases of SARS-CoV-2 infection.

All data were collected at the HUAV Lleida.

### Blood samples

Peripheral blood samples were directly collected in Blood Collection Tubes (BCTs), from QuantiFERON. They were for a SARS-CoV-2 research use only (RUO) assay, which was performed according to the manufacturer's recommendations (QIAGEN, Hilden, Germany). There were four different BCTs which contained the following antigens (Ag): Ag1 (an S1 subunit epitope of SARS-CoV-2 spike protein recognized by CD4 + T cells); Ag2 (S1 and S2 subunit epitopes of SARS-CoV-2 spike protein recognized by CD4 + and CD8 + T cells); a Mitogen tube (Mito), which was used as a positive control; and Nil, which was used as a negative control. The blood samples were incubated for 24 h at 37 °C. They were then centrifuged and plasma was stored at − 80 °C. IFN-γ levels for stimulated plasma were measured by enzyme-linked immunosorbent assay (ELISA), following the manufacturer’s instructions (www.quantiFERON.com). The Ag1 and Ag2 values were subtracted from the negative control value (Nil). The samples were run in triplicate and a standard curve was performed in each plate.

### Statistical analyses

All the statistical tests and graphs were performed using GraphPad Prism 6 (GraphPad Software). Quantitative variables were analysed using an ordinary two-way ANOVA test. The Fisher least significant difference test was used for multiple comparisons. Linear regression was tested to evaluate correlations between IFN-γ and age, EDSS, ALC and time from diagnosis. Fisher’s exact test was used to analyse changes in the percentage of responders in the contingency tables. The threshold for significance was set at 0.05.

## Results

### Description of participants

We analysed 159 DMT-treated pwMS to determine potential interactions of DMTs with the cellular immune response to the mRNA SARS-CoV-2 vaccinations. The clinical characteristics of the participants are described in Table 1.

**Table 1:** Description of the participants

	Total *N* (159)	Female *N* (%)	Mean age in years (range)	Mean time after diagnosis in years ± SD (range)	EDSS ± SD (range)	RRMS *N*	SPMS *N*	PPMS *N*	Undetermined *N*
OCRELIZUMAB	36	21 (58)	46 (21–68)	12 ± 7 (1–28)	3 ± 3 (0–7)	25	2	9	1
RITUXIMAB	13	9 (69)	51 (36–68)	12 ± 9 (1–29)	5 ± 2 (0–6.5)	1	9	2	1
FINGOLIMOD	14	10 (71)	49 (35–65)	15 ± 7 (4–26)	3 ± 3 (0–7.5)	9	5	0	0
NATALIZUMAB	25	21 (84)	42 (19–61)	14 ± 9 (1–30)	2 ± 2 (0–6)	22	2	1	0
GLATIRAMER ACETATE	4	2 (50)	42 (29–59)	8 ± 4 (4–14)	1 ± 2 (0–4)	4	0	0	0
ALEMTUZUMAB	8	5 (62)	42 (32–58)	15 ± 9 (5–26)	3 ± 2 (1–6.5)	7	1	0	0
DIMETHYL FUMARATE	26	16 (62)	43 (20–66)	12 ± 7 (1–30	1 ± 1 (0–2.5)	26	0	0	0
TERIFLUNOMIDE	22	16 (72)	48 (25–64)	17 ± 6 (1–24)	1 ± 2 (0–6.5)	20	2	0	0
CLADRIBINE	11	8 (73)	38 (27–61)	11 ± 10 (2–31)	0 ± 1 (0–4.5)	10	1	1	1

*SD* standard deviation

### Cellular immunity response to the booster dose

**Fig. 1:**
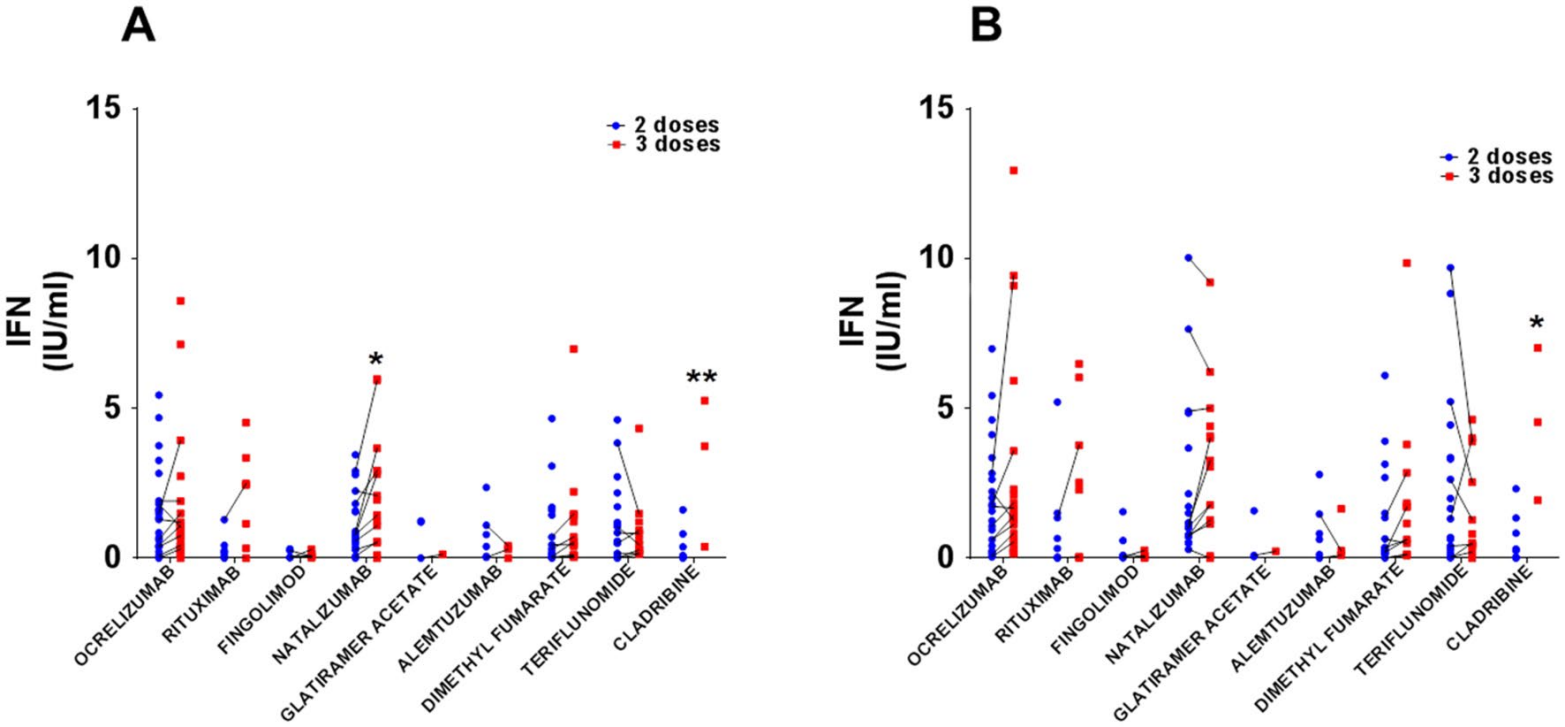
DMT interacts with cellular response to booster vaccination. **A** Employing Ag1 SARS-CoV-2 antigen, only natalizumab and cladribine-treated pwMS show higher IFN-γ production after booster vaccination. **B** Cladribine group increases IFN-γ after booster vaccination using Ag2 SARS-CoV-2 antigen. Fingolimod-treated pwMS do not develop cellular immune response even after booster vaccination. *p < 0.05, **p < 0.01 using Uncorrected Fisher’s LSD after 2way ANOVA considering the number of doses of vaccination and for each DMT group

We evaluated cellular immunity in our pwMS cohort using the QuantiFERON SARS-CoV-2 RUO kit. It was based on the IFN-γ response to spike protein using both the SARS-CoV-2 Ag1 and Ag2 tubes. We collected blood samples both before and after the patients received their third dose of vaccine. Comparing each DMT group individually, we noted that the booster dose only increased IFN-γ secretion in the case of natalizumab and cladribine stimulated with Ag1, while only cladribine was stimulated by Ag2. In addition, when we compared the response between the different groups after two doses, the fingolimod group showed a lower level of IFN-γ secretion than those of ocrelizumab, natalizumab and teriflunomide stimulated with either Ag1 or Ag2. Regarding differences for DMTs after three doses of vaccine, with Ag1 or Ag2 stimulation, the fingolimod response was weaker than those of ocrelizumab, natalizumab, cladribine and rituximab. The natalizumab group exhibited a greater response than those of alemtuzumab, dimethyl fumarate and teriflunomide after only Ag1 stimulation; the cladribine response was greater than that of alemtuzumab after Ag1 or Ag2 stimulation and also greater than those of dimethyl fumarate and teriflunomide after only Ag1 stimulation (Fig. 1A and B).

**Fig. 2:**
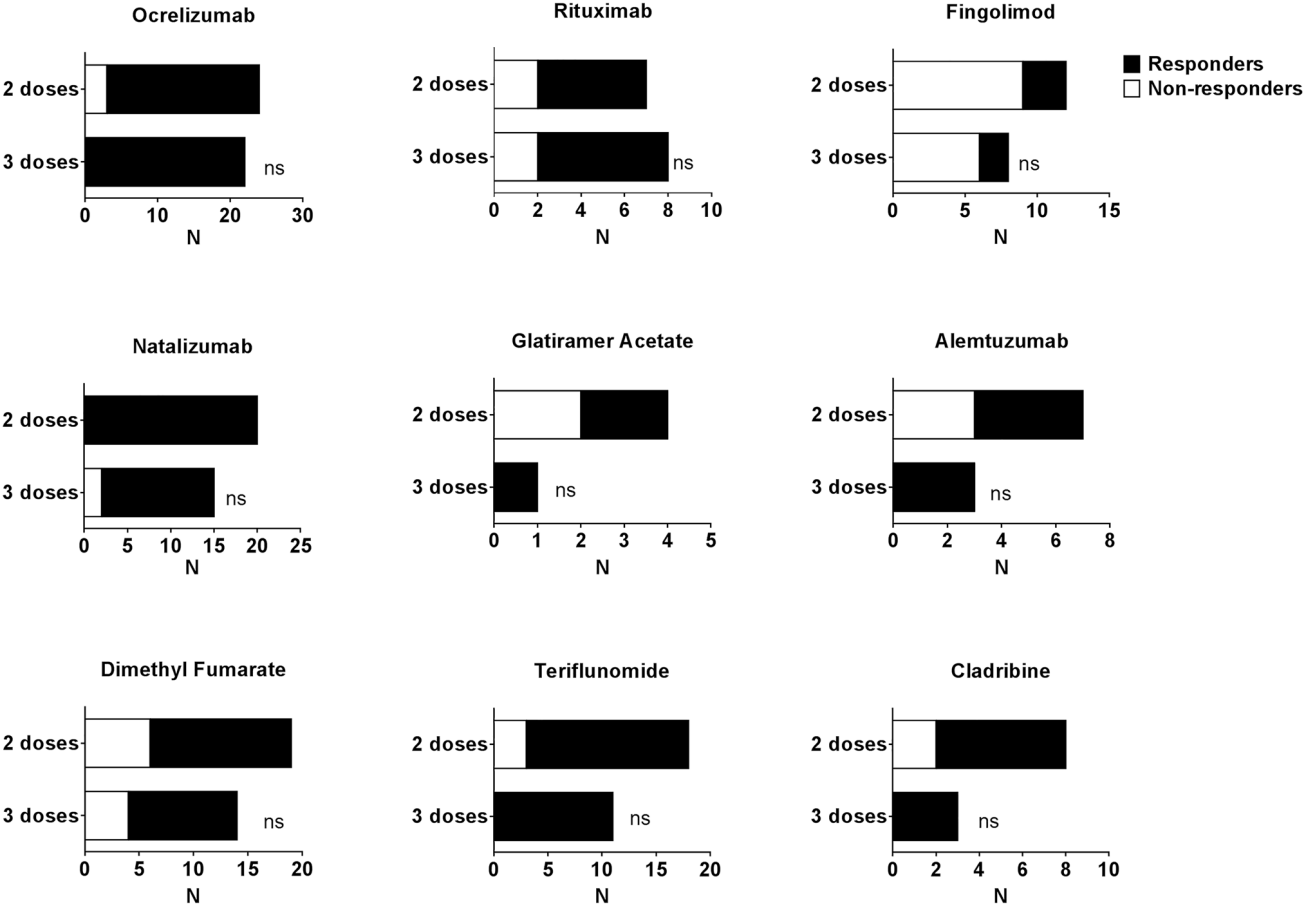
Booster vaccination does not alter response rate. All the DMT groups achieve maximum response ratios after two doses. Only fingolimod-treated pwMS have < 50% of responders. A response is considered positive when any of the Ag1 or Ag2 stimulation results are > 0.15 IU/ml of IFN-γ production. ns: not statistically significant according to Fisher’s exact test

To evaluate the proportion of vaccine responders, we set the threshold at 0.15 IU/ml, as previously described [19, 20] and considered cases as responders if any of their Ag1 or Ag2 values were higher than that level. The percentage of ocrelizumab responders was 87.5% after two doses (*N* = 23) and 100% after three doses (*N* = 22). The corresponding values for rituximab were 71.4% after two doses (*N* = 7) and 75% after three doses (*N* = 8); for fingolimod were 25% after two doses (*N* = 12) and 25% after three doses (*N* = 8), for natalizumab were 100% after two doses (*N* = 20) and 86.15% after three doses (*N* = 15), for glatiramer acetate were 50% after two doses (*N* = 4) and 100% after three doses (*N* = 1), for alemtuzumab were 57.1% after two doses (*N* = 7) and 100% after three doses (*N* = 3), for dimethyl fumarate were 68.4% after two doses (*N* = 19) and 71.4% after three doses (*N* = 14), for teriflunomide were 83.3% after two doses (*N* = 18) and 100% after three doses (*N* = 11), and for cladribine were 75% after two doses (*N* = 8) and 100% after three doses (*N* = 3) (Fig. 2).

### Impact of previous fingolimod treatment on cellular immune response

**Fig. 3:**
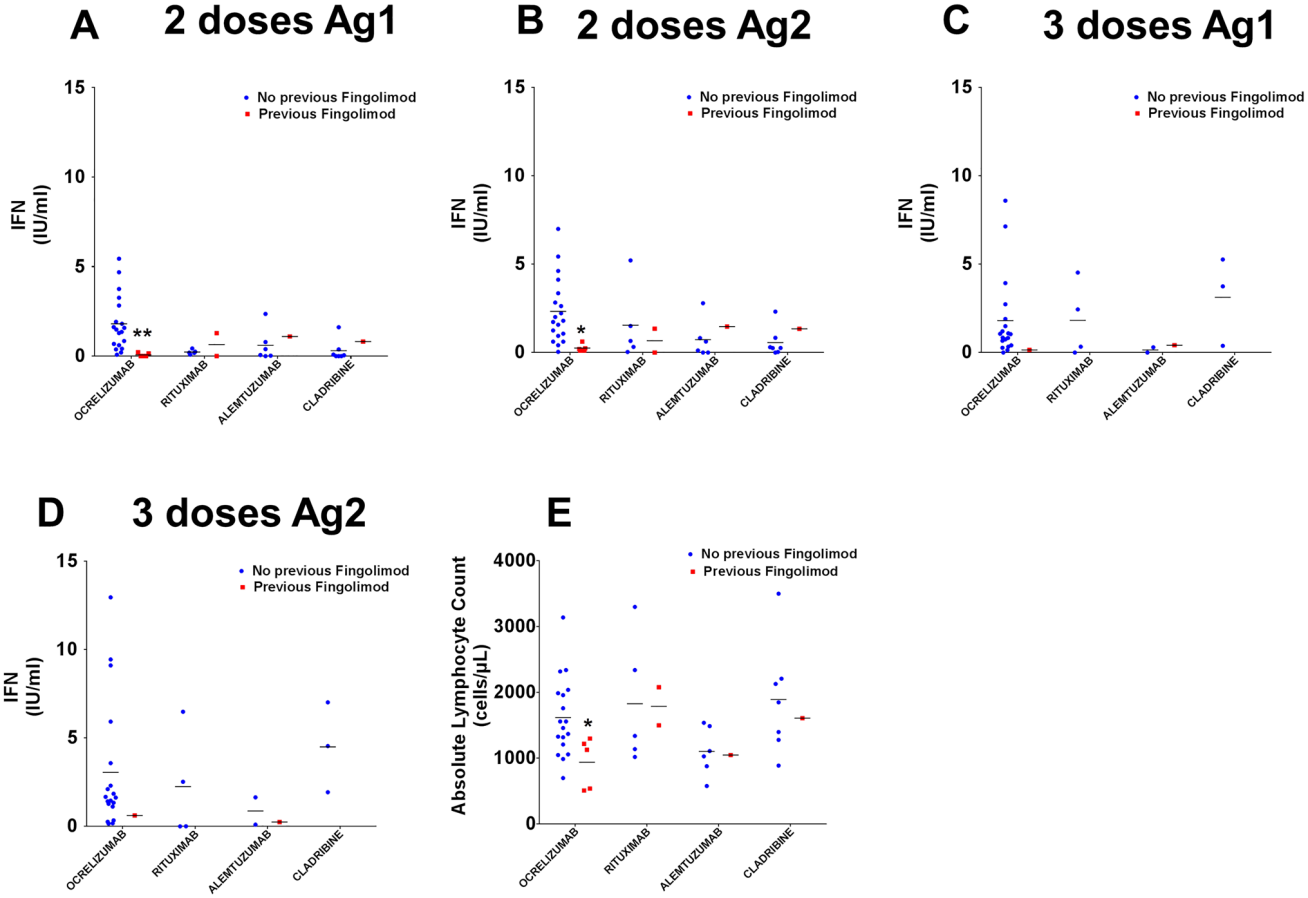
Previous fingolimod treatment hinders cellular response to vaccination. Ocrelizumab-treated pwMS who have had historical fingolimod treatment exhibit a reduced response to two doses of COVID-19 vaccination (**A**, **B**). Statistical analyses could not be performed after booster dose administration due to low N (**C**, **D**). Previous treatment with fingolimod produces a lower ALC in the ocrelizumab group (**E**): *p < 0.05 using Uncorrected Fisher’s LSD after two-way ANOVA, comparing previous vs no previous fingolimod treatment, for each DMT group

Taking fingolimod dramatically reduced the cell immunity response to COVID-19 vaccine. We analysed whether this effect persisted in pwMS who had previously received fingolimod treatments. After two doses of vaccine, participants currently being treated with ocrelizumab but who had previously been taking fingolimod (*N* = 4) showed lower levels of IFN-γ secretion after Ag1 (Fig. 3A) and Ag2 (Fig. 3B) exposure than those in the ocrelizumab group who had not previously taken fingolimod (*N* = 20). Statistical analyses could not be performed on other groups or for after they had received booster doses due to the low number of cases for both Ag1 (Fig. 3C) and Ag2 (Fig. 3D). To elucidate whether lymphopenia could be associated with a weaker response, we analysed ALC before patients received their first dose of vaccine. PwMS who had previously taken fingolimod who were in the ocrelizumab group had lower ALCs (Fig. 3E). We analysed the correlation between the time during which they had been taking ocrelizumab and until they received their second dose of vaccine and the associated T cell response (Ag1 and Ag2) and did not find any significant differences, but there was a positive trend for the *t* correlation: *R*^2^ = 0.8484, *p* value = 0.0789 for Ag1 and *R*^2^ = 0.7875, *p* value = 0.1126 for Ag2.

### Effect of SARS-CoV-2 infection on cellular immune response

**Fig. 4:**
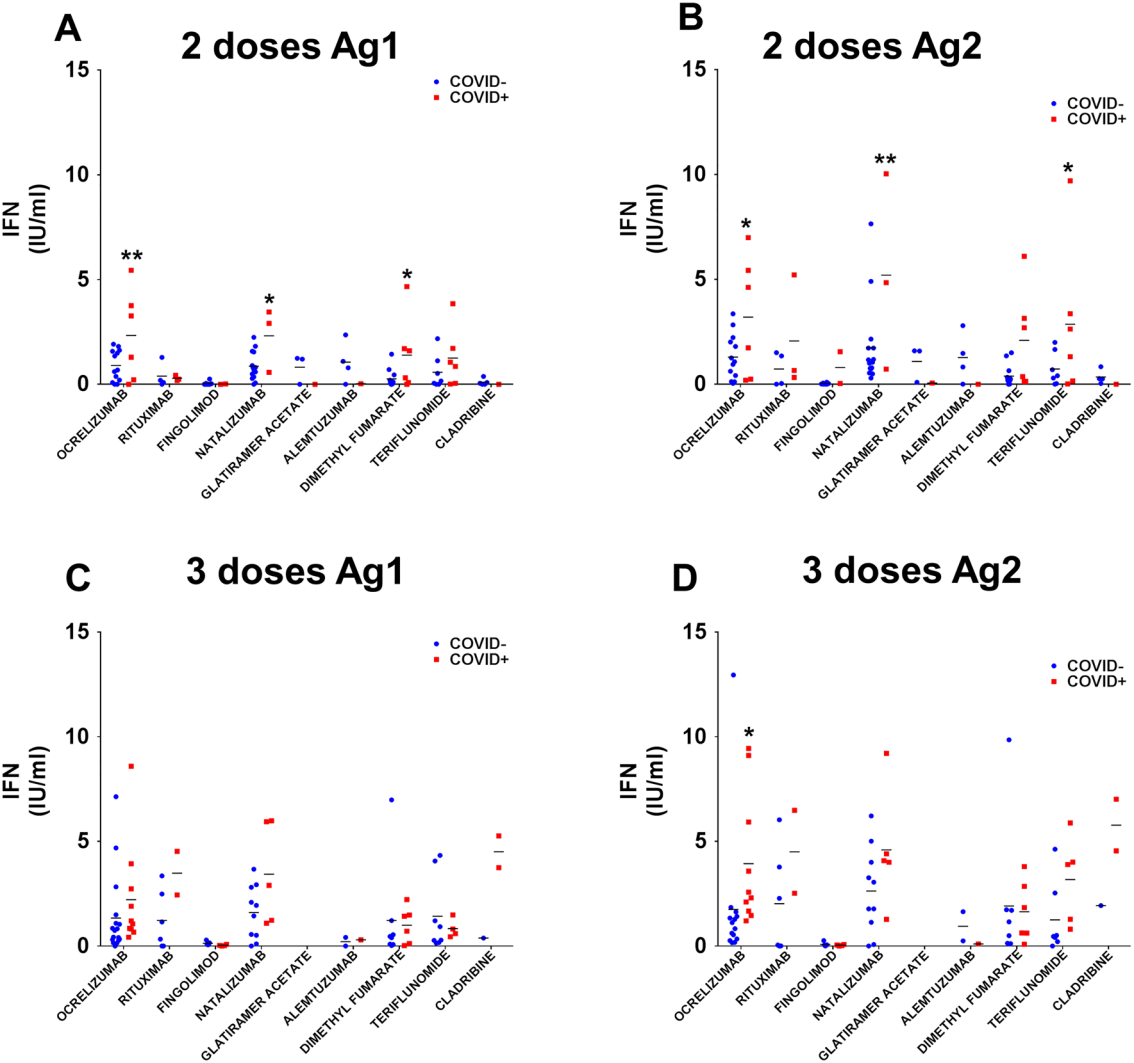
Previous SARS-CoV-2 infection increases cellular response after two doses of vaccine. Ocrelizumab, natalizumab and dimethyl fumarate-treated pwMS with confirmed SARS-CoV-2 infection and two doses of vaccine produces a better cellular response than those without previous infection for Ag1 exposure (**A**) and for natalizumab and Teriflunomide after Ag2 stimulation (**B**). However, previous COVID-19 only increases the cellular response after the booster vaccination in the case of ocrelizumab treatment (**C**, **D**). *p < 0.05, **p < 0.01 using Uncorrected Fisher’s LSD after two-way ANOVA, comparing COVID- with COVID + for each DMT group

We sought to analyse the effect of previous SARS-CoV-2 infection on cell immune response. We divided the DMT groups according to previous TAR/PCR confirmed infection prior to blood collection. Two vaccine doses and SARS-CoV-2 infection resulted in a higher level of IFN-γ secretion after Ag1 stimulus in ocrelizumab, natalizumab and dimethyl fumarate (Fig. 4A). In the case of Ag2 stimulus, natalizumab and teriflunomide exhibited higher IFN-γ in previous SARS-CoV-2 infected pwMS (Fig. 4B). Regarding the effect of three doses, no significant differences were observed in any of the DMT groups analysed for Ag1 (Fig. 4C) and only the ocrelizumab group with previous SARS-CoV-2 infection exhibited higher levels of IFN-γ upon Ag2 exposure (Fig. 4D).

### Correlation with clinical parameters and age

**Fig. 5:**
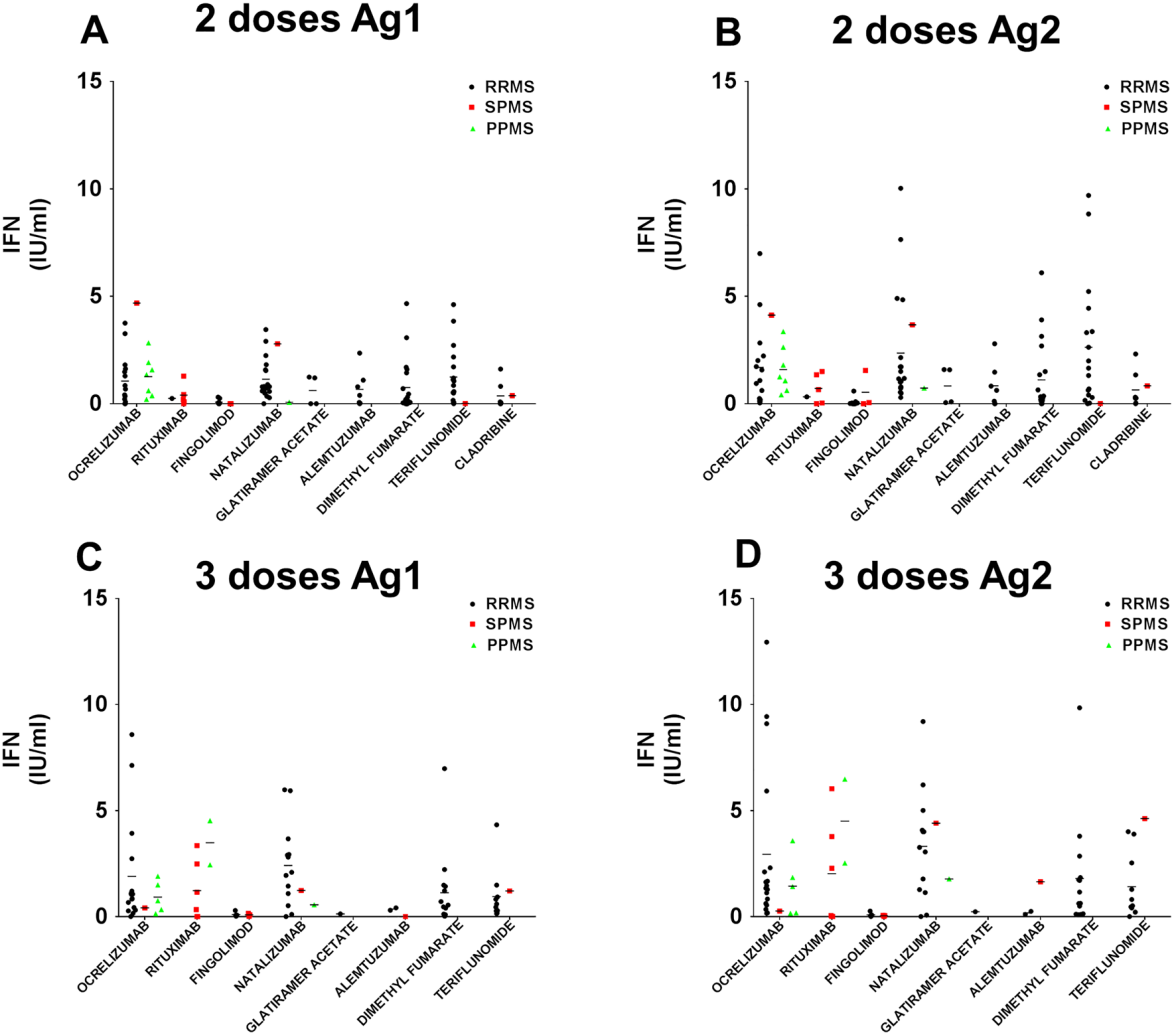
Clinical phenotype is not associated with cellular response. After two doses, clinical phenotype does not alter cellular response (**A**, **B**) and the booster effect is the same for all the clinical phenotypes in the DMT groups (**C**, **D**). No significant results are obtained using Uncorrected Fisher’s LSD after two-way ANOVA and comparing RRMS, PPMS and SPMS

We performed a correlation analysis between IFN-γ secretion (Ag1 and Ag2) and age, time after diagnosis, ALC and EDSS (Table 2). Age was positively correlated with Ag2 after three doses in the case of alemtuzumab. EDSS was negatively correlated with Ag2 after three doses in that of ocrelizumab. The time from diagnosis was negatively correlated with Ag1 and Ag2 after three doses in the cases of ocrelizumab and alemtuzumab treatment. ALC was positively correlated with Ag1 in the teriflunomide treatment after three doses of vaccine. The time between booster administration and analysis was positively correlated in the case of ocrelizumab. The vaccine cellular response was not influenced by the clinical phenotype after either two doses (Fig. 5A and B) or three doses (Fig. 5C and D).

**Table 2:** Multiple correlations with cellular response

Treatment	Two doses							Three doses						
	*N*	Ag1 R	Ag1 *p* value	Ag1 *p* value summary	Ag2 R	Ag2 *p* value	Ag2 *p* value summary	*N*	Ag1 R	Ag1 *p* value	Ag1 *p* value summary	Ag2 R	Ag2 *p* value	Ag2 *p* value summary
*Age*														
OCRELIZUMAB	23	0.200	0.361	ns	0.211	0.334	ns	22	− 0.337	0.125	ns	− 0.233	0.297	ns
RITUXIMAB	6	0.342	0.507	ns	0.406	0.425	ns	8	− 0.567	0.143	ns	− 0.632	0.093	ns
FINGOLIMOD	12	0.220	0.492	ns	0.368	0.239	ns	8	− 0.165	0.697	ns	− 0.640	0.088	ns
NATALIZUMAB	20	− 0.276	0.239	ns	0.029	0.903	ns	15	− 0.123	0.662	ns	− 0.247	0.376	ns
GLATIRAMER ACETATE	4	− 0.221	0.779	ns	− 0.208	0.792	ns	1	N/A	N/A	N/A	N/A	N/A	N/A
ALEMTUZUMAB	7	0.399	0.376	ns	0.362	0.425	ns	3	− 0.945	0.213	ns	1.000	**0.018**	*
DIMETHYL FUMARATE	19	− 0.277	0.251	ns	− 0.270	0.263	ns	14	− 0.153	0.601	ns	− 0.199	0.495	ns
TERIFLUNOMIDE	18	− 0.199	0.428	ns	− 0.259	0.300	ns	11	− 0.216	0.524	ns	− 0.356	0.283	ns
CLADRIBINE	8	− 0.239	0.568	ns	− 0.224	0.594	ns	3	− 0.123	0.922	ns	0.075	0.952	ns
*EDSS*														
OCRELIZUMAB	23	0.141	0.521	ns	− 0.057	0.796	ns	22.000	− 0.264	0.236	ns	− 0.430	**0.046**	*
RITUXIMAB	6	0.137	0.796	ns	0.243	0.642	ns	8.000	0.111	0.793	ns	0.074	0.871	ns
FINGOLIMOD	12	0.028	0.932	ns	0.076	0.813	ns	8.000	− 0.035	0.935	ns	− 0.048	0.892	ns
NATALIZUMAB	20	− 0.125	0.610	ns	− 0.056	0.821	ns	15.000	0.074	0.792	ns	− 0.107	0.675	ns
GLATIRAMER ACETATE	4	− 0.577	0.423	ns	− 0.563	0.437	ns	1.000	N/A	N/A	N/A	N/A	N/A	N/A
ALEMTUZUMAB	7	0.278	0.546	ns	0.296	0.519	ns	3.000	− 0.372	0.757	ns	1.000	0.333	ns
DIMETHYL FUMARATE	19	0.085	0.729	ns	0.165	0.499	ns	14.000	0.364	0.201	ns	0.193	0.524	ns
TERIFLUNOMIDE	18	− 0.298	0.230	ns	− 0.400	0.100	ns	11.000	− 0.238	0.481	ns	0.053	0.772	ns
CLADRIBINE	8	0.007	0.987	ns	0.083	0.846	ns	3.000	N/A	N/A	N/A	N/A	N/A	N/A
*Time from diagnosis*														
OCRELIZUMAB	23	− 0.101	0.656	ns	− 0.155	0.492	ns	22.000	− 0.461	**0.031**	*	− 0.412	0.057	ns
RITUXIMAB	6	0.546	0.263	ns	0.456	0.363	ns	8.000	− 0.067	0.875	ns	0.149	0.725	ns
FINGOLIMOD	12	0.162	0.616	ns	0.498	0.100	ns	8.000	− 0.538	0.213	ns	− 0.519	0.233	ns
NATALIZUMAB	20	− 0.173	0.467	ns	0.042	0.860	ns	15.000	− 0.159	0.571	ns	− 0.258	0.353	ns
GLATIRAMER ACETATE	4	0.775	0.225	ns	0.785	0.216	ns	1.000	N/A	N/A	N/A	N/A	N/A	N/A
ALEMTUZUMAB	7	0.154	0.741	ns	0.120	0.799	ns	3.000	0.946	0.211	ns	− 1.000	**0.020**	*
DIMETHYL FUMARATE	19	− 0.163	0.517	ns	− 0.145	0.565	ns	14.000	0.020	0.948	ns	− 0.046	0.881	ns
TERIFLUNOMIDE	18	0.138	0.586	ns	0.131	0.604	ns	11.000	0.196	0.564	ns	− 0.062	0.857	ns
CLADRIBINE	8	− 0.006	0.989	ns	0.081	0.849	ns	3.000	0.438	0.712	ns	0.606	0.586	ns
*ALC before booster dose*														
OCRELIZUMAB	23	0.243	0.264	ns	0.276	0.203	ns	22.000	0.348	0.113	ns	0.359	0.101	ns
RITUXIMAB	6	− 0.526	0.284	ns	− 0.183	0.729	ns	8.000	− 0.357	0.385	ns	− 0.309	0.457	ns
FINGOLIMOD	12	0.184	0.567	ns	− 0.079	0.806	ns	8.000	0.023	0.957	ns	0.172	0.685	ns
NATALIZUMAB	20	0.246	0.296	ns	0.139	0.559	ns	15.000	0.127	0.653	ns	0.128	0.649	ns
GLATIRAMER ACETATE	4	0.404	0.596	ns	0.383	0.617	ns	1.000	N/A	N/A	N/A	N/A	N/A	N/A
ALEMTUZUMAB	7	0.373	0.410	ns	0.409	0.362	ns	3.000	0.459	0.696	ns	− 0.115	0.927	ns
DIMETHYL FUMARATE	19	0.171	0.497	ns	0.232	0.354	ns	14.000	0.362	0.224	ns	0.367	0.218	ns
TERIFLUNOMIDE	18	0.289	0.246	ns	0.344	0.162	ns	11.000	0.629	**0.038**	*	− 0.394	0.231	ns
CLADRIBINE	8	0.396	0.332	ns	0.355	0.388	ns	3.000	0.886	0.307	ns	0.960	0.181	ns
*Booster follow-up period*														
OCRELIZUMAB								21	0.48	**0.03**	*	0.47	**0.03**	*
RITUXIMAB								7	0.27	0.56	ns	0.19	0.68	ns
FINGOLIMOD								6	0.57	0.24	ns	− 0.58	0.23	ns
NATALIZUMAB								15	− 0.15	0.60	ns	− 0.03	0.92	ns
GLATIRAMER ACETATE								1						
ALEMTUZUMAB								3	− 0.98	0.13	ns	0.99	0.10	ns
DIMETHYL FUMARATE								12	− 0.24	0.46	ns	− 0.19	0.55	ns
TERIFLUNOMIDE								11	− 0.12	0.73	ns	0.27	0.42	ns
CLADRIBINE								3	0.04	0.97	ns	− 0.15	0.90	ns
*COVID + follow-up period*														
OCRELIZUMAB	6	− 0.750	0.086	ns	− 0.829	0.058	ns							
RITUXIMAB	3	0.902	0.285	ns	1.000	0.333	ns							
FINGOLIMOD	2													
NATALIZUMAB	3	0.568	0.615	ns	0.500	> 0.9999	ns							
GLATIRAMER ACETATE	1													
ALEMTUZUMAB	1													
DIMETHYL FUMARATE	6	0.260	0.619	ns	0.486	0.356	ns							
TERIFLUNOMIDE	5	0.369	0.541	ns	0.200	0.783	ns							
CLADRIBINE	2													
*ALC before first dose*														
No previous fingolimod	18	0.195	0.439	ns	0.041	0.872	ns							
Previous fingolimod	5	0.601	0.283	ns	0.437	0.462	ns							
All	23	0.376	0.077	ns	0.250	0.250	ns							

^*Ag1* Antigen 1 from QuantiFERON SARS−CoV−2 Starter Set, *Ag2* Antigen 2 from QuantiFERON SARS−CoV−2 Starter Set, *ALC* Absolute Lymphocyte Count, *N/A* not available, *ns* non statistically significant. Significant values are highlighted in bold^

*p value< 0.05 for Pearson correlation test

## Discussion

A vaccination plan based on scientific evidence and balanced risk–benefit analysis is important for maintaining herd immunity and protecting the immunocompromised population. The COVID-19 pandemic is still on-going, as is periodic vaccination. The duration of the effect of humoral protection and nAb efficiency against new strains, the role of cell immunity, and the need for recurrent vaccination by pwMS are all a matter of concern in terms of public health. Describing cell immunity in pwMS who are taking DMTs and the risk factors that influence vaccine response can help to improve our decision-making regarding this illness.

Previous studies have shown a lack of humoral immunity response to COVID-19 vaccine in pwMS receiving anti-CD20 (rituximab and ocrelizumab), but appropriate cell immunity, given that the DMT only depletes B-cells (humoral response) [15–17]. However, it has been observed that treatment with fingolimod reduces both cell and humoral immunity [21]. Fingolimod prevents the egress of both B and T cells into secondary lymphoid organs, preventing them from reaching the location at which the vaccine is administered and triggering the immune response [22]. It has, however, been reported that a brief discontinuation of fingolimod administration can restore the humoral response, although not the cellular response, to the third dose of vaccine, even after an increase in the ALC [21]. Our results confirm these previous findings. Moreover, the reduction in cell immunity associated with fingolimod also persisted in pwMS currently being treated with ocrelizumab, even 2 years after their last administered dose of fingolimod. One plausible explanation for this would be a lower ALC just before receiving their first dose of vaccine. A recent report would support this hypothesis: it demonstrates a suppressed T-cell response to the booster dose after discontinuing the administration of fingolimod (2.5–4.4 months) [21]. Fingolimod could adversely affect the initial response to the vaccine and also that of subsequent booster doses. However, other factors may also influence the long-term effects of fingolimod on cellular immunity because there was no clear correlation between the ALC and IFN-γ. Specific T-cell count could offer a more precise way to correlate their presence with cellular immunity. Based on our data, the standard wash out period required to change from fingolimod to another DMT was not sufficient to allow the regeneration of cell immunity for a response to the mRNA vaccine. This is a previously undescribed risk factor for suboptimal responses to vaccines and one that should be taken into account for future pandemic control. Even so, it should be noted that the number of patients who switched from fingolimod to other DMTs was very limited; a larger cohort would be needed before these findings could be used to make recommendations. The existence of a positive trend between time on ocrelizumab after discontinuing treatment with fingolimod suggests the possible reversibility of this cellular immune response, although this could take a number of years. Quantitative data relating to cell immunity point to only a limited booster response for most DMT groups. However, after two doses of vaccine, all the DMT groups achieved the same response ratios as after three doses. The limitation on increasing IFN-γ production could also have implications for the severity of disease because this molecule has been associated with the milder COVID-19 [14]. Similar results had been obtained previously [22]. Nevertheless, the impact of the booster response was only analysed for patients treated with fingolimod and anti-CD20 after just 1 month of follow-up. Interestingly, in those cases, a different methodology was used, which further reinforces our results. In our case, we employed a faster and easier methodology which did not require the use of cell cytometry. Here, we highlight the importance of using a simple methodology so that more laboratories and hospitals can perform it. It may also be useful to personalize vaccination protocols and to make long-term follow-ups of cellular responses in different pwMS cohorts.

The cellular immune response was not influenced by the clinical phenotype under the ocrelizumab treatment. However, EDSS and time from diagnosis both negatively correlated with booster response (but not with responses to two doses of vaccine). This may highlight a negative impact of immunosenescence, associated with an increase in the degree of disability during the pathology [23]. Moreover, age was not correlated with T-cell response; this suggests that time from MS diagnosis could be more relevant than age alone for cell immune responses under ocrelizumab treatment. However, our cohort was not very old, so the older segment of the population was not very well represented. Furthermore, cell immune response did not correlate with age, EDSS, and time from diagnosis in other DMT groups. Clinical phenotype was not relevant for triggering a cellular immune response. As a result, further risk stratification related to clinical phenotype in pwMS would not be necessary. The lack of ALC correlation and cell response associated with all the DMTs, except teriflunomide, pointed to the remaining T cells being sufficient in number to produce a cellular immune response. However, critical T-cell lymphopenia induced by fingolimod inhibited cellular immune response and this effect persisted for more than 2 years. There are also other, more selective S1PR modulators, that have been approved for MS treatment (ozanimod, ponesimod and siponimod) and could overcome this issue. Some of them have a faster rate of lymphocyte reconstitution after withdrawal (ponesimod) and could play a different role at this level [24].

SARS-CoV-2 vaccines had side effects that could potentially increase disability in pwMS or trigger a relapse of the disease [25–27]; this is, however, a controversial topic [28]. For this reason, some patients have already rejected a fourth dose of vaccine. Moreover, most of them are still protected and do not need it. Booster doses with other vaccines are generally administered after first verifying a lack of adequate humoral response after primary vaccination. This has not, however, been the case with regard to the policy used in relation to COVID-19 and risk groups, including pwMS receiving DMTs. Repeated vaccination and/or re-infection with novel vaccine-resistant strains can potentially trigger T-cell and vaccine exhaustion even in people with an otherwise adequate level of immunization, resulting in a loss of efficacy [29, 30]. The possibility of adding cellular response analyses to anti-S antibody surveys could help us to identify the people who most require booster doses. Such a policy is, however, unfeasible in many countries because of the economic impact that it could have and so risk–benefit evaluations should be studied by their health authorities.

There are currently several new antiviral therapeutic alternatives that can be used against SARS-CoV-2 infection (including remdesivir, nirmatrelvir/ritonavir and molnupiravir) that can be administered according to prioritization criteria; pwMS treated with anti-CD20 or fingolimod would fall within this category. Evusheld (tixagevimab/cilgavimab) is also indicated for promoting passive immunity for pre-exposure prophylaxis of COVID-19 in MS patients taking DMTs and who are not protected against SARS-CoV-2 (anti SARS-CoV-2 S < 260 BAU). These options can modify the risk of patient infection and prognosis. These novel therapies, combined with information about cellular and humoral responses in pwMS, should be integrated into a management algorithm for protecting pwMS treated with DMTs. In conclusion, cellular response is preserved in most pwMS, except for those treated with fingolimod, or with ocrelizumab after previous fingolimod treatment. This suggests that an alternative way of protecting them should be studied. Two doses are enough to achieve a sufficient response ratio in pwMS which can be maintained over time. This, however, raises doubts about the need for rapid revaccination.

## Data Availability

Data are available on reasonable request. The data that support the findings of this study are available from the corresponding author on reasonable request.
